# Estrogen promotes stemness and invasiveness of ER-positive breast cancer cells through Gli1 activation

**DOI:** 10.1186/1476-4598-13-137

**Published:** 2014-06-03

**Authors:** Ying Sun, Yunshan Wang, Cong Fan, Peng Gao, Xiuwen Wang, Guangwei Wei, Junmin Wei

**Affiliations:** 1 Department of Chemotherapy, Cancer Center, Qilu Hospital, Shandong University, 107 Wenhua Xi Road, Jinan 250012, China; 2 Department of Human Anatomy and Key Laboratory of Experimental Teratology, Ministry of Education, Shandong University School of Medicine, 44 Wenhua Xi Road, Jinan Shandong 250012, China; 3 International Biotechnology R&D Center, Shandong University School of Ocean, 180 Wenhua Xi Road, Weihai, Shandong 264209, China; 4 Department of Pathology, Shandong University School of Medicine, 44 Wenhua Xi Road, Jinan, Shandong 250012, China

**Keywords:** Sonic hedgehog, Shh, Epithelial-mesenchymal transition, Epithelial-mesenchymal transition, Estrogen

## Abstract

**Background:**

Although long-term estrogen (E2) exposure is associated with increased breast cancer (BC) risk, and E2 appears to sustain growth of BC cells that express functional estrogen receptors (ERs), its role in promoting BC stem cells (CSCs) remains unclear. Considering that Gli1, part of the Sonic hedgehog (Shh) developmental pathway, has been shown to mediate CSCs, we investigated whether E2 and Gli1 could promote CSCs and epithelial-mesenchymal transition (EMT) in ER^+^ BC cell lines.

**Methods:**

We knocked down *Gli1* in several BC cells using a doxycycline-controlled vector, and compared *Gli1*-knockdown cells and *Gli1*^+^ cells in behavior and expression of ER, Gli1, ALDH1 (BC-CSC marker), Shh, Ptch1 (Shh receptor) and SOX2, Nanog and Bmi-1 (CSC-associated transcriptions factors), using PCR; tissue microarrays, western blot; chromatin immunoprecipitation q-PCR, confocal immunofluorescence microscopy; fluorescence-activated cell sorting; annexin–flow cytometry (for apoptosis); mammosphere culture; and colony formation, immunohistochemistry, Matrigel and wound-scratch assays.

**Results:**

Both mRNA and protein expressions of ER correlated with those of Gli1 and ALDH1. E2 induced Gli1 expression only in ER^+^ BC cells. E2 promoted CSC renewal, invasiveness and EMT in ER^+^/Gli1^+^ cells but not in *Gli1*-knockdown cells.

**Conclusions:**

Our results indicate that estrogen acts via Gli1 to promote CSC development and EMT in ER^+^ BC cells. These findings also imply that Gli1 mediates cancer stem cells, and thus could be a target of a novel treatment for ER^+^ breast cancer.

## Background

Serial studies have suggested that breast cancer stem cells (CSCs) play critical roles in tumor growth, invasion, metastasis and resistance to cytotoxic agents and radiation [1]. CSCs, like other stem cells, have unique characteristics, such as the ability to self-renew, generate differentiated cells [2] and express key stemness-associated transcription factors, including SOX2, Nanog and Bmi-1 [3-5]. In human breast cancer, CSCs also appear to be enriched within cell subpopulations with a CD44^+^/CD24^-/low^ surface marker profile or with high intracellular aldehyde dehydrogenase 1 (ALDH1) activity [6-8]. However, the molecular mechanisms regulating breast CSC frequency, localization and maintenance remain poorly understood.

Epithelial-mesenchymal transition (EMT) is a process that involves epithelial cells acquiring a mesenchymal phenotype and migratory capability and plays an important role in tumor metastasis [9,10]. Importantly, the EMT process has been shown to be associated with the acquisition of stem cell properties in normal and cancer cells [11,12]. This crucial event results from transcriptional repression of E-cadherin through overexpression of several different EMT-inducing factors, such as Snail, vimentin and Bmi-1 [5,13]. The link between EMT and CSCs enables cancer cells to migrate from the primary tumor and colonize at distant sites.

Estrogen is vital for normal postpubertal mammary development and long-term exposure to estrogens is proven to be associated with an increased risk of breast cancer. Although abundant evidence has suggested that estrogen sustains the growth of breast cancer cells expressing functional estrogen receptors (ERs) [14], the role of estrogen in promoting breast CSCs remains controversial. Some researchers think that ER-positive cells contribute to the stem cell compartment directly stimulated by hormones [15,16], while others consider that estrogen may stimulate the expansion of a specific stem cell compartment in a paracrine manner [17-19]. Similar to the dynamic spatio-temporal signaling that governs the specification and maintenance of normal mammary gland stem cells [18,20], specific hormone-growth factor paracrine signaling pathways have also proven to regulate stem-like breast cancer cells [17,18].

Sonic hedgehog (Shh)/Gli signaling controls a variety of developmental processes, such as pattern formation, differentiation, proliferation and organogenesis. The net effect of pathway stimulation by Shh protein binding to the protein patched homolog 1 receptor (Ptch1) is the activation of members of the Gli family of zinc finger transcription factors, which translate the extracellular Shh stimulus into defined transcriptional programs in a context-dependent and cell type-specific manner [21]. Gli1, a member of the Gli family, was originally identified as an amplified gene in malignant glioma [22] and was proven to be able to regulate stem cells and cancer stem cells [23]. Moreover, Gli1 can be expressed without active Shh signaling [24]. However, whether Gli1 is involved in estrogen-induced stemness and invasiveness in breast cancer remains uninvestigated.

In this study, we investigated whether estrogen could promote CSC maintenance and EMT through Gli1 in human ER-positive breast cancer cell lines MCF-7 and HCC1428, and ER-negative cell lines BT549 and MDA-MB-231. Here, we provide direct evidence that estrogen promoted the acquisition of the CSC phenotype and EMT through transcriptional activation of Gli1 only in ER-positive breast cancer cells.

## Results

### ER expression is positively correlated with Gli1 and ALDH1 in human breast cancer cell lines

**Figure 1:**
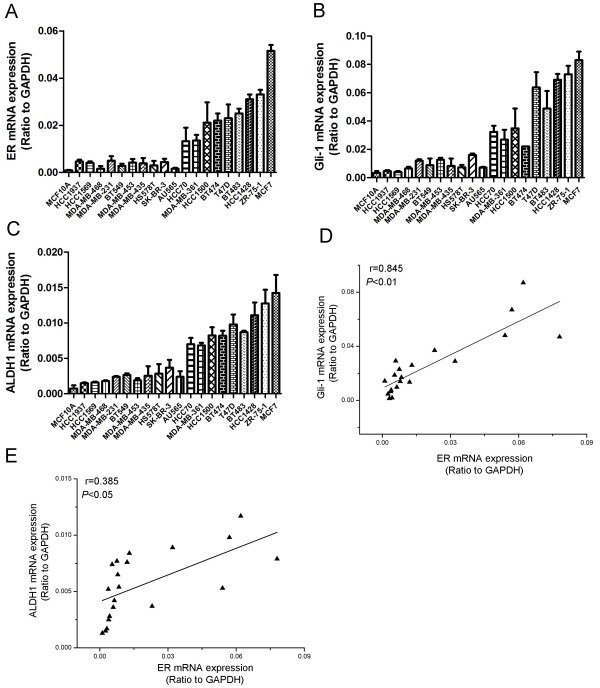
**Endogenous expression of ER, Gli1 and ALDH1 in human breast cancer cells lines.** MRNA levels of **(A)***ER*, **(B)***Gli1* and **(C)***ALDH1* were measured using real-time RT-PCR. **(D & E)** Linear correlation assays were used to analyze the relationship between ER and Gli1 **(D)** or ER and ALDH1 **(E)** expression levels.

**Figure 2:**
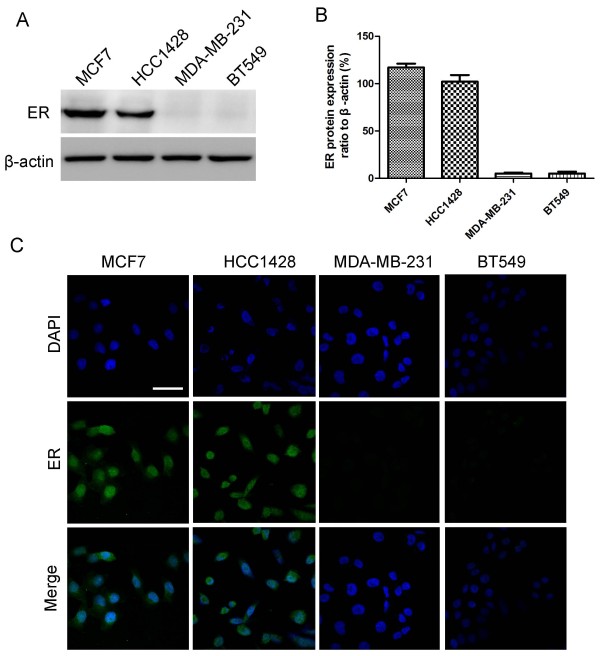
**ER expression in MCF-7, HCC1428, MDA-MB-231 and BT549 cells. (A)** ER protein levels were analyzed using western blotting. β-Actin levels were measured as a loading control. **(B)** Histograms illustrate ER protein expression relative to that of β-actin. All data corresponded to the mean ± SD of three independent experiments. **(C)** Immunofluorescence staining of ER in MCF-7, HCC1428, MDA-MB-231 and BT549 cells. Green represents ER staining. Blue signals represent nuclear DNA staining with DAPI. Scale bars indicate 25 μm.

We examined the endogenous mRNA expression of breast cancer stem cell markers *ALDH1*, *Gli1* and *ER* in several human breast cancer cell lines using real-time polymerase chain reaction (PCR; Figure 1A–C). Our data showed that *ALDH1* and *Gli1* mRNAs were detectable in all cell lines. We then used linear correlation analysis to evaluate the relationship among *Gli1*, *ALDH1* and *ER* expression levels. We found that *ER* expression positively correlated with *Gli1* and *ALDH1* (Figure 1D & E). Next, we examined the expression of the ER protein using western blotting and immunofluorescence assays in MCF-7, HCC1428, MDA-MB-231 and BT549 cells. As shown in Figure 2A–C, ER expression was higher in MCF-7 and HCC1428 cells and barely detectable in MDA-MB-231 and BT549 cells.

### Estrogen-induced Gli1 expression only in ER-positive breast cancer cells

**Figure 3:**
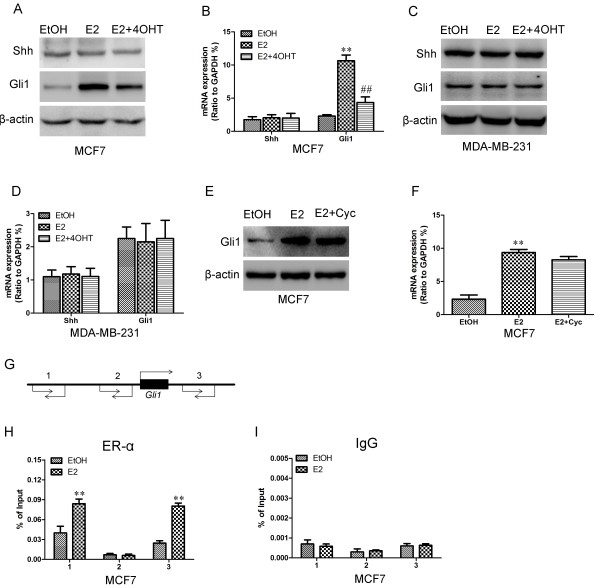
**Estrogen promoted the expression of Gli1 through noncanonical Shh signaling in MCF-7 cell lines. (A & C)** Western blotting was used to detect **(A)** Gli1 and Shh expression in MCF-7 or **(C)** MDA-MB-231 cells incubated with 10 nM estrogen (E2) with or without 1 μM 4-hydroxy tamoxifen (4OHT) for 4 days. β-Actin was used as a loading control. In **(B)** MCF-7 or **(D)** MDA-MB-231 cells, mRNA expression levels of *Gli1* and *Shh* were measured using qRT-PCR, and expression was normalized to that of GAPDH. **(E)** Western blotting was used to detect Gli1 protein expression in E2 and/or cyclopamine-treated MCF7 cells. **(F)** RT-PCR was used to detect *Gli1* mRNA expression in E2 and/or cyclopamine-treated MCF7 cells. **(G)** Schematic presentation of three regions relative to the *Gli1* transcriptional start site used to design primers to test for ER-α occupied abundance. **(H)** QChIP was performed to assess ER-α occupancy in ETOH and E2-treated MCF7 cells. **(I)** IgG was used as negative control. “% of input” indicates the ratio of DNA fragment of each promoter region bound by ER-α to the total amount of input DNA fragment without ER-α antibody pull-down. All data correspond to the mean ± SD of three independent experiments. **, ## indicate significant differences from the control (*p* < 0.001).

Because ER expression was correlated with Gli1, we then asked whether estrogen could influence Shh pathway activation in breast cancer cells. MCF-7, HCC1428, MDA-MB-231 and BT549 cells were incubated with 10 nM estrogen (E2) with or without 1 μM 4-hydroxy tamoxifen (4OHT) for 4 days, after which Shh and Gli1 protein and mRNA expression were measured. In ER-positive MCF-7 and HCC1428 cells, Gli1 expression was significantly increased in estrogen-treated cells compared with that in control (ETOH-treated) cells. Additionally, 4OHT inhibited estrogen-induced expression of Gli1 (Figure 3A, B & Additional file 1: Figure S1A). However, E2 failed to significantly increase Gli1 expression in ER-negative MDA-MB-231 and BT549 cells (Figure 3C, D & Additional file 1: Figure S1B). Shh expression was not affected in any of the four cell lines tested. Our results indicated that estrogen activated the Shh/Gli1 pathway only in ER-positive breast cancer cells through noncanonical Shh signaling.To elucidate the mechanism by which E2 activated the Shh/Gli1 pathway, we tested cyclopamine, a canonical inhibitor of Smo, in the Shh signaling pathway. Cyclopamine plus E2 were incubated with MCF-7 cells for 4 days. We then analyzed and compared Gli1 protein and mRNA expression levels in ETOH and E2-treated cells. Cyclopamine did not inhibit estrogen-induced activation of Gli1 (Figure 3E & F).

We also treated breast cancer cells with the Shh ligand to examine the effect of Shh on Gli1 and Ptch1 mRNA expression. Addition of various concentrations of Shh to these cells for 24 h increased both Gli1 and Ptch1 mRNA expression levels relative to untreated cells (Additional file 2: Figure S2). These results indicated that Gli1 activation was not mediated by canonical Shh signaling. Given that E2 modulated Gli1 transcription, quantitative chromatin immunoprecipitation (qChIP) assays were performed in ETOH and E2-treated MCF7 cells to determine the mechanism of this E2 effect. We found increased ER protein binding to the *Gli1* promoter (area #1), as well as to the *Gli1* gene body (area #3) in E2-treated MCF7 cells compared with ETOH-treated control cells (Figure 3H). The occupancy of IgG at the *Gli1* gene promoter was not changed by E2 treatment (Figure 3I). These results indicated that E2 induced transcriptional activation of *Gli1,* probably through enriching ER occupancy at the *Gli1* gene promoter and gene body.

### Estrogen-induced CSC survival and self-renewal in ER-positive breast cancer cells is mediated by Gli1

**Figure 4:**
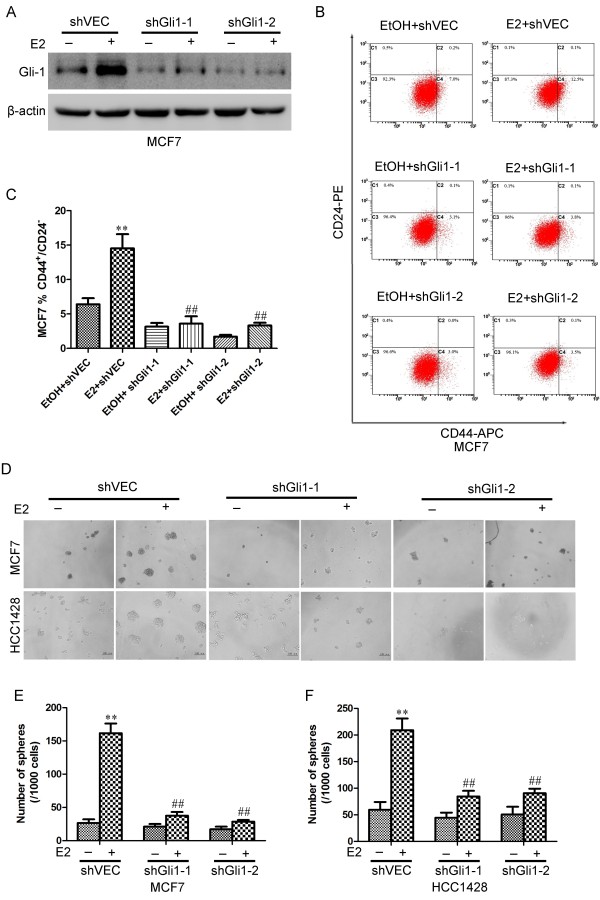
**Knockdown of Gli1 in ER-positive breast cancer cells inhibited estrogen induced sphere formation and CD44**^**+**^**/CD24**^**-/low **^**cells. (A)** MCF-7 cells were transfected with control shRNA (shVEC) or shGli1 (shGli1-1 or shGli1-2) in the absence or presence of E2. Gli1 protein levels were analyzed using western blotting. β-Actin levels were measured as a loading control. **(B)** MCF-7 cells were treated with 10 nM E2 or ETOH as a control and transfected with shGli1-1, shGli1-2 or shVEC. After 4 days, cells were stained with anti-CD44-APC and anti-CD24-PE antibodies, and CD44^+^/CD24^-/low^ subpopulations were examined using flow cytometry. **(C)** Histograms illustrate percentages of CD44^+^/CD24^-/low^ subpopulations. **(D)** Representative images of MCF-7 and HCC1428 cells in the absence or presence of E2. MCF-7 and HCC1428 cells (1 cell/μL) were cultured in 96-well plates containing 100 μL SFM in each well with or without E2 for seven days. **(E & F)** The number of spheres was counted under a microscope. Error bars represent SEMs. **, ## indicate significant differences from the control (*p* < 0.001).

To determine whether Gli1 mediated E2-induced stemness and invasiveness in breast cancer cell lines, MCF-7 and HCC1428 cells were transfected with pSingle vectors carrying short hairpin RNA (shRNA) targeting Gli1. Cells were grown in the absence (shVEC transfected cells) or presence (shGli1-1 and shGli1-2 transfected cells) of Dox for 4 days. They were then harvested and analyzed using western blotting. As shown in Figure 4A and Additional file 1: Figure S1C, Gli1 protein levels in shGli1-1 and shGli1-2 cells were significantly reduced when compared with those of the control (shVEC).

Cells were then transfected with shVEC, shGli1-1 or shGli1-2 (the latter two representing Gli1-knockdown cells) and treated with E2 for 4 days, following which the CD44^+^/CD24^–/low^ cell population was observed using flow cytometry. We found that treatment of shVEC cells with E2 induced a statistically significant expansion of CD44^+^/CD24^–/low^ stem-like cells. In Gli1-knockdown cells, E2 failed to significantly increase the proportion of CD44^+^/CD24^–/low^ cells (Figure 4B, C and Additional file 1: Figure S1D & E). The parallel expression patterns of Gli1 and CSC markers indicated that Gli1 may regulate the expansion of CSCs in estrogen-treated ER-positive breast cancer cells (Figure 4A–C & Additional file 1: Figure S1C–E).

Next, shVEC and Gli1-knockdown cells were cultured at very low densities (1 cell/μL) in 96-well plates containing serum-free medium with or without E2 for seven days. In the presence of E2, shVEC cells produced more and larger spheres compared with control cells (*p* < 0.01; Figure 4D–F). In Gli1-knockdown cells, E2 failed to significantly increase the number and size of these spheres (*p* < 0.01; Figure 4D–F). The significant inhibition in Gli1-knockdown cells of the estrogen-mediated increase in sphere formation suggested that Gli1 was indeed involved in the regulation of estrogen-induced self-renewal of breast CSCs.

**Figure 5:**
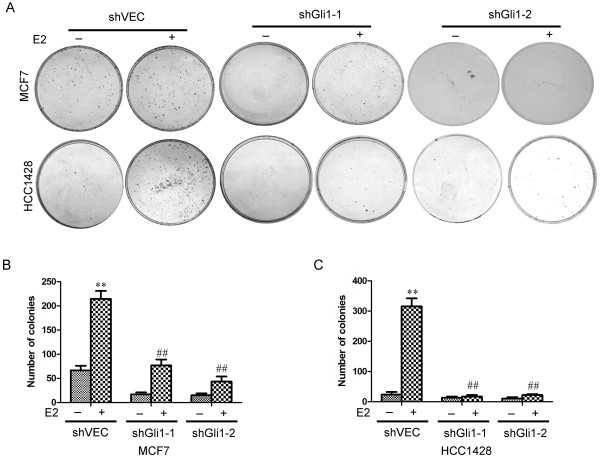
**Estrogen increased the proliferation of ER-positive breast cancer cells. (A)** Colony formation assays in MCF-7 and HCC1428 cells. MCF-7 and HCC1428 cells were transfected with vehicle control shRNA (shVEC), shGli1-1 or shGli1-2 in the absence or presence of E2. **(B & C)** The number of colonies was counted under a microscope. Error bars represent the SEM. **, ## indicate significant differences from the control (*p* < 0.001).

**Figure 6:**
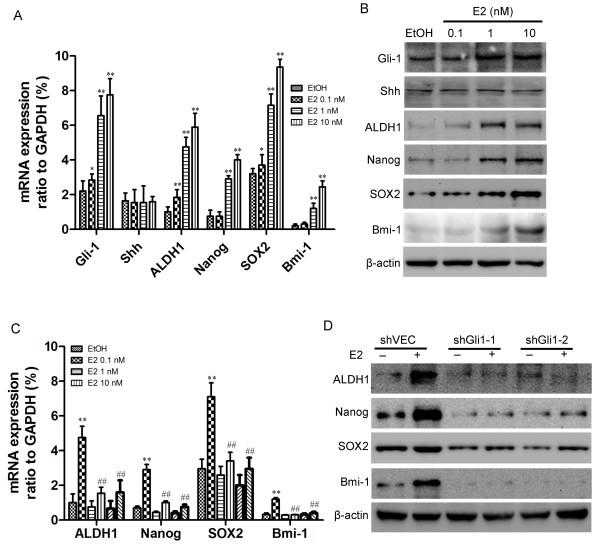
**E2 enhanced the expression of CSC markers in ER-positive MCF-7 cells through Gli1. (A)** MCF7 cells were treated with various concentrations (0.1, 1 or 10 nM) of E2 for 4 days. MRNA expression levels of *Gli1*, *Shh*, *ALDH1*, *Nanog*, *SOX2* and *Bmi-1* were measured using qRT-PCR, and expression was normalized to that of GAPDH. **(B)** Western blotting was used to determine the effects of various concentrations of E2 on the expression of Gli1, Shh, ALDH1, Nanog, SOX2 and Bmi-1 proteins in MCF-7 cells. β-Actin was used as a loading control. **(C)** MCF-7 cells were transfected with vehicle control shRNA (shVEC), shGli1-1 or shGli1-2 and grown in medium with ETOH or E2 for 4 days. RNAs were extracted and assayed using qRT-PCR to determine the expression levels of *ALDH1*, *Nanog*, *SOX2* and *Bmi-1* mRNAs. Bars represent the means ± SEs of three experiments (**, ##: *P* < 0.01). **(D)** Effects of E2 on the expression of ALDH1, Nanog, SOX2 and Bmi-1 were estimated using western blotting. β-Actin was used as a loading control.

The proliferative potential of these breast cells under the same conditions were also assessed using a colony formation assay. We observed that shVEC cells showed a significant increase in the number and size of colonies in the presence of E2. However, E2-induced proliferation was not observed in Gli1-knockdown cells (Figure 5A–C). Flow cytometric analyses of apoptosis and cell cycle progression further confirmed the role of Gli1 in E2-induced ER-positive breast cancer cell survival and proliferation (Additional file 3: Figure S3).To further investigate the mechanism through which Gli1 regulated CSCs, MCF7 cells were treated with E2 (0–10 nM) for 4 days. Gli1, Shh, ALDH1, Nanog, SOX2 and Bmi-1 expression levels were measured using real-time PCR and western blotting. As shown in Figure 6A and B, Gli1, ALDH1, Nanog, SOX2 and Bmi-1 expression levels all gradually increased with increasing E2 concentrations, whereas Shh was virtually unchanged. The expression of CSC markers was also measured following transfection of MCF-7 cells with shVEC or Gli1-knockdown constructs and subsequent treatment with E2. As expected, ALDH1, Nanog, SOX2 and Bmi-1 expression levels were significantly enhanced in shVEC cells after E2 treatment, but remained unchanged in Gli1-knockdown cells following E2 treatment (Figure 6C & D). Because E2 simultaneously induced CSC-related gene expression and Gli1 activation without affecting Shh expression, these results implied that E2 induced the expansion of breast CSCs through a noncanonical ligand-independent Shh/Gli signaling pathway.

### Estrogen-induced EMT in breast cancer cells is mediated by Gli1

**Figure 7:**
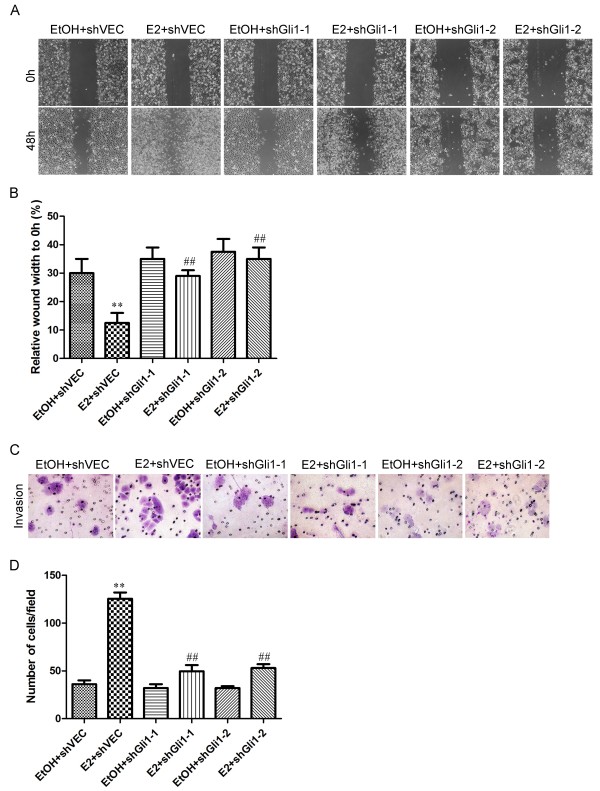
**E2 enhanced the invasiveness of MCF-7 cells via Gli1. (A)** Representative images of wounds at 0 and 48 h in the presence of ETOH or E2. **(B)** Histograms illustrate relative wound widths at 0 and 48 h. The migration distance of each cell was measured after the photographs were converted to Photoshop files. **(C)** Matrigel invasion assay. MCF-7 cells were seeded into Matrigel-coated invasion chambers and were treated with ETOH (control) or E2 for 48 h. Representative images of stained invaded cells are shown. Magnification 100×. **(D)** The number of migrated cells was quantified by counting cells from 10 random fields. Data are representative of three independent experiments. Bars represent the means ± SEs of three experiments (**, ##: *P* < 0.01).

**Figure 8:**
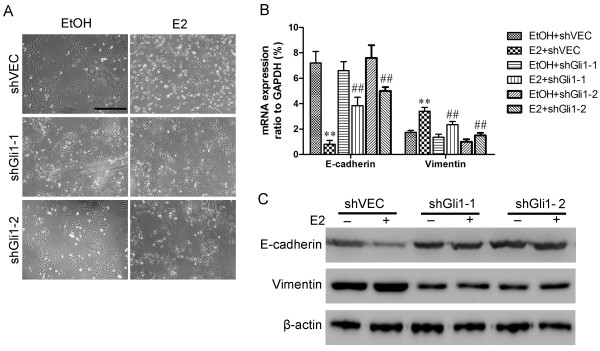
**Knockdown of Gli1 inhibited estrogen-induced EMT in MCF-7 cells. (A)** Estrogen induced morphological change from an epithelial to a fibroblast-like appearance in shVEC-transfected MCF-7 cells. Gli1-knockdown cells (shGli1-1 and shGli1-2) still maintained their epithelial morphologies seven days after treatment with E2. Scale bar,200 μm. ShVEC, shGli1-1 and shGli1-2-transfected MCF-7 cells were cultured in medium with 1% FBS in the presence ETOH or E2 for seven days. **(B)** Real-time qRT-PCR was used to determine the expression levels of E-cadherin and vimentin mRNAs. Bars represent the means ± SEs of three experiments (**, ##: *P* < 0.01). **(C)** Western blotting was used to determine the effects of shGli1 on the expression level of E-cadherin and vimentin proteins. β-Actin was used as a loading control.

Recent data has indicated that cancer cells often undergo EMT during cancer metastasis, which results in a mesenchymal fibroblast-like morphology, reduced intercellular adhesion, increased motility and increased invasive and migratory properties [25]. EMT-type tumor cells are closely associated with tumor recurrence and therapeutic resistance and display several characteristics of CSCs [26]. Firstly, we investigated whether estrogen-treated breast cancer cells exhibited Gli1-dependent changes in cell motility using a wound healing assay in MCF-7 and HCC1428 cells. An area devoid of cells was created at time 0 h by scraping the monolayer of shVEC-transfected and Gli1-knockdown cells, followed by incubation of the cells with ETOH or E2 for 48 h. Compared with Gli1-knockdown cells, estrogen-treated shVEC-transfected MCF-7 and HCC1428 cells showed a significantly higher rate of migration (*p* < 0.01), and the leading edges along the scraped areas had almost coalesced 48 h after scraping (Figure 7A, B and Additional file 4: Figure S4A & B). A Matrigel invasion assay was also performed. The results showed that the number of E2-treated shVEC-transfected cells that migrated across both the Matrigel and the insert were 3.23 and 1.97 times higher than those of ETOH-treated MCF-7 and HCC1428 cells, respectively (Figure 7C, D and Additional file 4: Figure S4C & D). In Gli1-knockdown cells, E2 failed to alter the number of migrated or invaded cells. These data indicated that Gli1 played a significant role in mediating the invasiveness of ER-positive breast cancer cells.Next, we examined the impact of Gli1 knockdown on estrogen-induced EMT. As shown in Figure 8A, MCF-7 cells transfected with shGli1-1 and shGli1-2 exhibited an epithelial phenotype in the presence of E2, similar to that displayed by MCF-7 cells in the absence of E2, regardless of whether they were transfected with shGli1-1, shGli1-2 or shVEC. In contrast, MCF-7 cells transfected with shVEC displayed a spindle-shaped morphology after E2 stimulation. This morphological transformation was consistent with the reduction in E-cadherin expression and increase in vimentin expression in estrogen-treated shVEC-transfected cells (Figure 8B & C). In Gli1-knockdown cells, Gli1 knockdown partly reversed E2-induced expression of E-cadherin and vimentin, as shown using real-time PCR and western blot analysis (Figure 8B & C). This observation was consistent with the notion that Gli1 may be responsible for the estrogen-induced EMT in ER-positive breast cancer cells.

### Correlation of ER, Gli1 and ALDH1 expression in human breast cancer tissues

**Figure 9:**
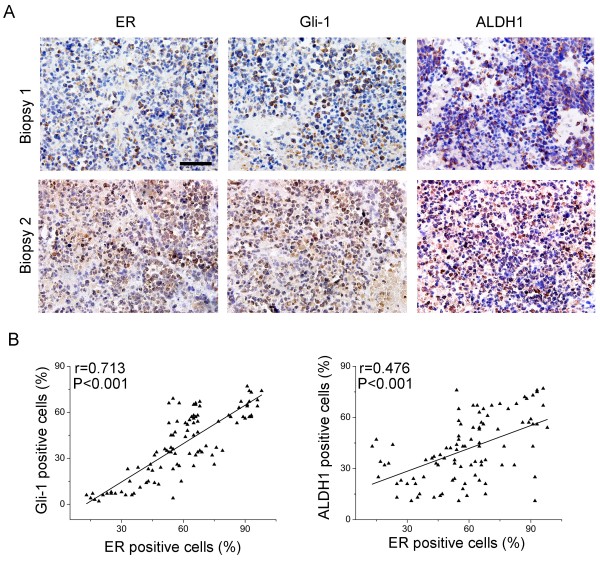
**The expression of ER correlated with the expression of EMT and stem cell-related markers in breast cancer tissue. (A)** Expression levels of ER, Gli1 and ALDH1 were measured in a breast cancer tissue microarray using immunohistochemistry. Final magnification, 40×. Scale bar, 50 μm. **(B)** ER expression was significantly correlated with Gli1 and ALDH1 expression in breast cancer tissues. The coordinate axis equals the percentage of positive cells relative to the total number of cancer cells.

To determine whether there were any correlations among ER, Gli1 and the CSC marker ALDH1 in breast cancer specimens, we used a tissue microarray containing 100 breast cancer samples and 10 adjacent normal breast tissue or adenosis samples to analyze the expression of ER, Gli1 and ALDH1 using immunohistochemical staining. Gli1 and ALDH1 protein expression levels were low or undetectable in the 10 normal samples (data not shown) but comparatively high in breast tumor tissues (Figure 9A). Moreover, ER expression was positively correlated with Gli1 and ALDH1 expression levels (Figure 9B).

## Discussion

The incidence of breast cancer is increasing worldwide, and breast cancer is quickly becoming one of the most serious diseases for women today. Currently, treatments for breast cancer mainly include chemotherapy, surgery, endocrine and radiation therapies. However, metastases and recurrences have become the major bottlenecks in all of these treatment options. The CSC hypothesis has shed new light on the development of resistance to therapy, proposing that a pool of malignant cells with stem cell-like properties exists and that this pool of malignant cells increases the capacity for resistance to common chemotherapeutic treatments as compared with their more differentiated nontumorigenic counterparts; these CSCs are therefore thought to be responsible for tumor recurrence after treatment [9]. Thus, therapeutic strategies that specifically target breast CSCs can be effective in eradicating tumors and reducing the risk of relapse and metastasis.

Estrogens are essential mitogens for normal mammary epithelial cells, and prolonged exposure to estrogen is considered an important risk factor for breast cancer development. Although 60–70% of human breast cancers are ER-positive [27] and respond to anti-estrogen therapy, many of them will inevitably progress to the estrogen-independent stage and develop resistance to anti-estrogen therapy [14]. The existence of CSCs offers a simple explanation as to the presence of endocrine resistance in breast cancer [28-30]. There have been conflicting reports about the effects of estrogen on breast CSCs; some studies have reported that estrogen can increase or decrease CSC numbers in breast cancer cell lines [18,31]. Here, we identified estrogen as an important positive modulator of CSC properties in ER-positive breast cancer cell lines.

Emerging studies have demonstrated that CSC-enriched populations exhibit low/no expression of ER in ER-positive cell lines [17,18,32], suggesting that the effects of estrogen on breast CSCs are not direct. Our data described here demonstrated that breast CSC activity and cell surface marker expression were increased by estrogen exposure through its effects on the estrogen/Gli1-signaling axis. We found that estrogen induced transcriptional activation of *Gli1* through enriching ER occupancy at the *Gli1* gene promoter and gene body. It may represent a novel mechanism underlying this phenomenon, which we will try to address in future studies. Moreover, because cyclopamine inhibited Smo, which is a critical component of the canonical pathway, but did not inhibit the expression of Gli1, our current data strongly suggested the involvement of a noncanonical pathway.

EMT, a process through which epithelial cells acquire characteristics of mesenchymal cells, is thought to play an important role in invasion and metastasis [33] and is closely associated with the “stemness” of cancer cells [12,34]. Our experiments demonstrated that estrogen induced cellular morphological changes in breast cancer cells, consistent with the acquisition of the EMT phenotype, as characterized by the upregulation of vimentin and downregulation of E-cadherin expression. EMT was significantly decreased when Gli1 was knocked down, and this was associated with reduced CSC numbers, as indicated in the mammosphere assay, and accompanied by decreased expression of Gli1. These data indicated that Gli1 mediated the estrogen-induced EMT in ER-positive breast cancer cells.

ER-negative tumors, especially triple-negative breast cancer (TNBC), are more aggressive and tend to harbor more CSCs [35]. While much of our data were collected using the ER-positive MCF7 and HCC1428 cell lines, we were able to observe upregulation of Gli1 in many different primary human tumor tissue samples (Figure 1B), suggesting the relevance of this pathway in primary tumors. Our results showed that the expression levels of Shh and Gli1 were both high in MDA-MB-231 and BT549 cells; however, the expression of these targets was not affected by estrogen. Goel *et al.* showed that a novel autocrine pathway involving vascular endothelial growth factor (VEGF)/VEGF receptor neuropilin-2 (NRP2), α6β1 and Gli1 contributed to the initiation of TNBC and demonstrated that Gli1 was activated in noncanonical Shh signaling [35]. Additionally, DiMeo *et al.* provided evidence of a molecular link between Wnt signaling, self-renewal, EMT and metastasis in basal-like breast cancers [36].

Taken together, the data presented in our study provided evidence that Shh signaling pathway components were widely expressed in breast cancer. Estrogen had the potential to promote the viability of breast CSCs via binding to the Gli1 promoter in ER-positive breast cancer *in vitro*. It will be important to also test whether these results can be translated in animal studies. A critical property of CSCs is their ability to repopulate heterogeneous tumor populations and to functionally demonstrate tumor-initiating capacity *in vivo*. There is significant interest in understanding the biology behind solid tumor stem cells and identifying drug targets and therapeutic approaches for eliminating these tumor subpopulations.

## Conclusion

CSC self-renewal and EMT enable tumor metastasis, a key driving force for tumor growth and recurrence. Estrogen is a steroid hormone that has been closely linked to enhanced growth and invasion of breast cancer. Using breast cancer cells lines (MCF-7 and HCC1428), we demonstrated that estrogen may act via Gli1 to promote CSC development and EMT in ER-positive breast cancer cells, which may contribute to breast tumor malignancy. Thus, Gli1, which promotes the expansion of cancer stem cells, may be a potential therapeutic target for novel treatments for ER-positive breast cancer.

## Methods

### Chemicals and antibodies

Lipofectamine 2000 transfection reagent and TRIzol LS Reagent were purchased from Invitrogen (Grand Island, NY, USA). The DAB substrate kit for peroxidase was purchased from Vector Laboratories, Inc. (Burlingame, CA, USA). Antibodies against Gli1, ERα, E-cadherin, Vimentin, Nanog, Bmi-1, SOX2, Smo and β-actin were from Cell Signaling Technology (Danvers, MA, USA). Anti-ALDH antibodies were from BD (Franklin Lakes, NJ, USA). Anti-Shh antibodies were from Santa Cruz Biotechnology (Santa Cruz, CA, USA). Amino-terminal Shh (ShhN) peptide was from R&D Systems (Minneapolis, MN, USA). Unless otherwise noted, all other chemicals were from Sigma (St. Louis, MO, USA).

### Cell culture

All the human breast cancer cell lines were purchased from the American Type Culture Collection (ATCC, Manassas, VA, USA). HCC1428, MDA-MB-231, BT549, HCC1937, HCC1569, HCC70, HCC1500, HS578T, SK-BR-3, AU565 and ZR-75-1 cells were grown in RPMI 1640 medium supplemented with 10% fetal bovine serum (FBS) and 1% penicillin/streptomycin. MCF-7, T47D, BT474, BT483, MDA-MB-468, MDA-MB-453, MDA-MB-435 and MDA-MB-361 cells were grown in DMEM medium supplemented with 10% FBS and 1% penicillin/streptomycin. MCF-10A cells were cultured in DMEM/F12 supplemented with 20 ng/mL epidermal growth factor, 0.01 mg/mL insulin, 500 ng/mL hydrocortisone, 5% heat-inactivated HS, 100 IU/mL penicillin and 100 μg streptomycin. All the cell lines were grown at 37°C in an atmosphere containing 5% CO_2_ and 95% air.

### Gli1-specific shRNA inhibition

To knock down Gli1 expression, shRNA targeting Gli1 expressed in the pSingle vector was prepared as described previously [37]. Cells were grown in culture dishes until they reached 75% confluence, at which point they were transfected for 24 h with pSingle-shRNA specific to Gli1 using the Lipofectamine 2000 transfection reagent according to the manufacturer’s instructions. The tight on/off regulation of the pSingle vector system and coordinate inactivation of the target gene was mediated using doxycycline (Dox). Expression of the shRNA in the absence of induction was extremely low and prevented unwanted suppression of the target gene. When Dox was added to the culture medium, transcriptional suppression was relieved, permitting the shRNA to be transcribed. After transfection, cells were trypsinized, collected and subjected to various experiments.

### Real-time RT-PCR

Total RNA was extracted from different cell lines using the TRIzol reagent. Quantitative determination of RNA levels was performed in triplicate in three independent experiments. Real-time PCR and data collection were performed on the ABI PRISM 7900HT sequence detection system (Applied Biosystems, Foster City, CA, USA). The housekeeping gene *GAPDH* was used as an internal control to normalize the expression levels of different genes. Quantification of the relative expression of target genes was performed using the ΔΔCt method. The following gene-specific primers were used: ERα, forward 5′-AGA TGG TCA GTG CCT TGT TGG-3′ and reverse 5′-CCA AGA GCA AGT TAG GAG CAA ACA G-3′; Shh, forward 5′-GTG TAC TAC GAG TCC AAG GCA C-3′ and reverse 5′-AGG AAG TCG CTG TAG AGC AGC-3′; Gli1, forward 5′-GCG ATC TGT GAT GGA TGA GAT TCC C-3′ and reverse 5′-TGC CTT GTA CCC TCC TCC CGA A-3′; SOX2, forward 5′-GCT GTA TGG CTG CTG CAC TTC A-3′ and reverse 5′-GCA CAC GCA CCC AGC ACT GT-3′; Nanog, forward 5′-AAT ACC TCA GCC TCC AGC AGA TG-3′ and reverse 5′-TGC GTC ACA CCA TTG CTA TTC TTC-3′; Bmi-1, forward 5′-GAC CAC TAC TGA ATA TAA GG-3′ and reverse 5′-CAT TTG TCA GTC CAT CTC TC-3′; ALDH1, forward 5′-GTT AGC TGA TGC CGA CTT GG-3′ and reverse 5′-CCC ACT CTC AAT GAG GTC AAG-3′; Ptch1, forward 5′-TCG CTC TGG AGC AGA TTT CC-3′ and reverse 5′-TCT CGA GGT TCG CTG CTT TT -3′; and GAPDH, forward 5′-CAA GGT CAT CCA TGA CAA CTT TG-3′ and reverse 5′-GTC CAC CAC CCT GTT GCT GTA G-3′.

### Western blot analysis

Cells were lysed in lysis buffer (1% Triton X-100, 150 mM NaCl, 10 mM Tris–HCl [pH 7.4], 1 mM EDTA, 1 mM EGTA, 2 mM NaF, 1 mM sodium orthovanadate, 10 μg/mL leupeptin, 10 μg/mL pepstatin, 10 μg/mL aprotinin, 10 μg/mL E 64 and 1 mM Pefabloc; EMD). Protein concentrations were determined using an Enhanced BCA Protein Assay Kit (Beyotime Institute of Biotechnology, Jiangsu, China) and then boiled for 5 min. Protein samples (30 μg) were separated by sodium dodecyl sulfate-polyacrylamide gel electrophoresis (SDS-PAGE) and transferred to polyvinylidene difluoride (PVDF) membranes (Millipore Corporation, Billerica, MA, USA). Membranes were rinsed in Tris-buffered saline containing Tween 20 (TBST), blocked with 5% bovine serum albumin (BSA) for 2 h at room temperature, and incubated with the primary antibody at 4°C overnight. The membranes were then rinsed and incubated in peroxidase-conjugated secondary antibodies for 1 h at room temperature. After washing, proteins were detected using enhanced chemiluminescence (ECL) (Millipore Corporation). Membranes were stripped and reprobed with anti-β-actin mouse monoclonal antibodies to confirm equal loading of samples.

### Confocal immunofluorescence microscopy

Cell lines were plated on culture slides (Costar, Manassas, VA, USA). After 4 days, cells were rinsed with phosphate-buffered saline (PBS), fixed with 4% paraformaldehyde in PBS, and permeabilized using 0.5% Triton X-100. Cells were then blocked for 30 min in 10% BSA (Sigma Aldrich, St. Louis, MO, USA) in PBS and then incubated with primary monoclonal antibodies in 10% BSA overnight at 4°C. After three washes in PBS, slides were incubated for 1 h in the dark with FITC-conjugated secondary goat anti-mouse or goat anti-rabbit antibodies (Invitrogen). After three additional washes, slides were stained with 4-,6-diamidino-2-phenylindole (DAPI; Sigma Aldrich) for 5 min to visualize the nuclei and examined using a Carl Zeiss confocal imaging system (LSM 780; Carl Zeiss, Jena, Germany).

### Fluorescence-activated cell sorting (FACS) analysis

Anti-CD44-APC and anti-CD24-PE antibodies used for FACS analysis were obtained from Biolegend (San Diego, CA, USA). Briefly, for each cell line, 1 × 10^6^ cells were aliquoted into two tubes; tube 1 was stained with IgG isotype controls for APC and PE, and tube 2 was stained with anti-CD44-APC and anti-CD24-PE antibodies. Cells were incubated with the appropriate antibodies for 30 min on ice and then washed with PBS. Cells were analyzed using a FACSCalibur flow cytometer (BD Biosciences); each sample required 10,000 cells for analysis.

### Cell cycle assays

Aliquots of 1 × 10^5^ cells were collected using trypsinization and treated with 50 μg/mL DNase-free RNase and 20 μg/mL propidium iodide (PI) following the manufacturer’s instructions. Cells were analyzed using an FC500 instrument (Beckman Coulter, Brea, CA) with MultiCycle for Windows software (Beckman Coulter) for detailed cell cycle status.

### Apoptosis analysis

Apoptosis was determined using the Annexin V-FITC Apoptosis Detection Kit (BD Biosciences Pharmingen, San Diego, CA, USA) according to the manufacturer’s instructions. Briefly, cells were detached and resuspended in 100 μL binding buffer containing FITC-Annexin V and PI. After incubation for 15 min at room temperature in the dark, cells were analyzed using an FC500 instrument (Beckman Coulter). Annexin V-positive cells were classified as apoptotic.

### Mammosphere culture

Mammosphere culture was performed as described by Dontu *et al.* with slight modifications [38]. Single-cell suspensions were plated in ultralow attachment 96-well plates (Costar) at different densities of viable cells. Cells were grown in serum-free mammary epithelial growth medium (MEGM), supplemented with 1:50 B27 (Invitrogen), 20 ng/mL epithelial growth factor (EGF), 20 ng/mL basic fibroblast growth factor (bFGF; BD) and 10 μg/mL heparin (Sigma). The number of spheroids was counted after 7–10 days. For *in vitro* propagation, primary spheres were collected, dissociated into single-cell suspensions and plated in ultralow attachment 96-well plates. The secondary number of spheroids was counted 14 days after plating.

### Colony formation assay

Cells were seeded in triplicate at 500 cells/6-cm dish in complete medium. After 3 weeks of growth, cells were fixed and stained with crystal violet (0.1% w/v in 20 nM 4-morpholinepropanesulfonic acid; Sigma), and visible colonies were counted according to the number of cells in each colony. All experiments were repeated at least three times. Plating efficiency was determined as the number of colonies formed divided by the total number of cells plated.

### Wound scratch migration assay

Cells were seeded in 6-cm culture dishes, and cell monolayers were wounded by scratching with sterile plastic 200-μL micropipette tips and photographed using phase-contrast microscopy immediately following and 48 h after wounding. Migration assays were independently performed in triplicate. The migration distance of each cell was measured after the photographs were converted to Photoshop files.

### Matrigel invasion assay

Invasion of cells was measured in Matrigel (BD)-coated transwell inserts (6.5 mm, Costar) containing polycarbonate filters with 8-μm pores, as detailed previously [39]. Inserts were coated with 50 μL of 1 mg/mL Matrigel matrix according to the manufacturer’s recommendations. A total of 2 × 10^5^ cells in 200 μL serum-free medium were plated in the upper chamber, and 600 μL of medium containing 10% FBS was added to the lower chamber. After 24 h incubation, top cells (noninvasive) were removed, and bottom cells (invasive) were counted. Cells that invaded to the lower surface of the membrane were fixed in 4% paraformaldehyde and stained with 0.5% crystal violet. For each membrane, five random fields were counted at 10× magnification. Data were presented as the mean ± SD from three independent experiments performed in triplicate.

### Chromatin immunoprecipitation (ChIP)-qPCR

A chromatin immunoprecipitation kit (Cat. 17–371) was purchased from Millipore and ChIP experiments were carried out essentially as described (27). Immunoprecipitated DNA was analyzed on an ABI PRISM 7900HT sequence detection system. Primers used for detection of promoters following ChIP are located at -653 to -472 bp (1), -297 to -116 bp (2) and +167 to +359 bp (3) of the *Gli1* promoter.

### Tissue microarrays and immunohistochemical analyses

Tissue microarrays of breast samples (BC081116a) were from Alenabio (Xian, China). The manufacturer provided clinical and pathological information. Immunostaining was performed using the avidin-biotin-peroxidase complex method (UltrasensitiveTM, MaiXin, Fuzhou, China). Sections were deparaffinized in xylene, rehydrated in a graded series of alcohols and then boiled in 0.01 M citrate buffer (pH 6.0) for 2 min in an autoclave. Hydrogen peroxide (0.3%) was applied to block endogenous peroxide activity, and sections were incubated with normal goat serum to reduce nonspecific binding. Tissue sections were incubated with rabbit polyclonal anti-Gli1 antibodies (1:100 dilution), mouse monoclonal anti-ER antibodies (1:50 dilution) or mouse monoclonal anti-ALDH1 antibodies (1:100 dilution). Staining for these antibodies was performed at room temperature for 2 h. Biotinylated goat anti-mouse serum IgG was used as a secondary antibody. After washing, sections were incubated with streptavidin-biotin conjugated with horseradish peroxidase and the peroxidase reaction was developed with 3,30-diaminobenzidine tetrahydrochloride. Two independent, blinded investigators examined the slides randomly. Five views were examined per slide and 100 cells were observed per view at 400× magnification.

### Statistical analysis

Data were described as the mean ± SD. Associations between protein expression levels in the breast tissue microarray were assessed using Spearman’s rank correlation test. Comparisons between different groups were carried out using the Student’s two-tailed t-test. The limit of statistical significance was set at a *P-value* <0.05. Statistical analysis was performed using SPSS/Win11.0 software (SPSS, Inc., Chicago, IL, USA).

## Abbreviations

CSC: Cancer stem cell; EMT: Epithelial-mesenchymal transition; ALDH1: Aldehyde dehydrogenase activity; ER: Estrogen receptor; Shh: Sonic hedgehog; Ptch1: Protein patched homolog 1; Smo: Smoothened; 4OHT: 4-hydroxy tamoxifen; E2: Estrogen; shRNA: Short hairpin RNA; Dox: Doxycycline; FBS: Fetal bovine serum; PVDF: Polyvinylidene difluoride; TBST: Tris-buffered saline containing Tween 20; BSA: Bovine serum albumin; ECL: Enhanced chemiluminescence; PBS: Phosphate-buffered saline; FACS: Fluorescence-activated cell sorting; EGF: Epithelial growth factor; bFGF: Basic fibroblast growth factor; MEGM: Mammary epithelial growth medium.

## Competing interests

The authors declare that they have no competing interests.

## Authors’ contributions

JMW, GWW, and YSW designed the experiments. YS and CF performed the experiments. XWW and PG performed the statistical analysis. YS, JMW and GWW wrote the manuscript. All authors approved the final draft of this manuscript.

## Supplementary Material

Additional file 1: Figure S1 Estrogen promoted the expression of Gli1 and CSCs in HCC1428 cells. (A & B) Western blotting was used to detect Gli1 in HCC1428 (A) and BT549 (B) cells treated with 10 nM estrogen (E2) with or without 1 μM 4-hydroxy tamoxifen (4OHT) for 4 days. β-Actin was used as a loading control. (C) HCC1428 cells were transfected with control shRNA (shVEC), shGli1-1, or shGli1-2 in the absence or presence of E2. Gli1 protein levels were analyzed by western blotting, and β-actin levels were measured as a loading control. (D) HCC1428 cells were treated with 10 nM E2 or ETOH (control) and transfected with shGli1-1, shGli1-2, or shVEC. After 4 days, cells were stained with anti-CD44-APC and anti-CD24-PE antibodies, and CD44^+^/CD24^-/low^ subpopulations were examined by flow cytometry. (E) Histograms illustrating the percentage of CD44^+^/CD24^-/low^ subpopulations. All data correspond to the mean ± SD of three independent experiments. **, ## indicate significantly different from the control, *p* < 0.001. Click here for file

Additional file 2: Figure S2 Effects of Shh on Ptch1 and Gli1 mRNA expression in breast cancer cells. Shh was added at the specified levels to serum-starved breast cancer cells for 24 h, after which total RNA was extracted and subjected to qRT-PCR analysis for Ptch1 and Gli1 mRNAs expression levels. qRT-PCR was used to detect Ptch1 in MCF7 (A), HCC1428 (B), MDA-MB-231 (C), and BT549 (D). qRT-PCR was used to detect Gli1 in MCF7 (E), HCC1428 (F), MDA-MB-231 (G), and BT549 (H). All data correspond to the mean ± SD of three independent experiments. *indicate significantly different from the control, *p* < 0.05. **indicate significantly different from the control, *p* < 0.01. Click here for file

Additional file 3: Figure S3 Effects of E2 on the cell cycle and apoptosis of shVEC- and shGli1-transfected MCF-7 cells. MCF-7 cells were transfected with control shRNA (shVEC), shGli1-1, or shGli1-2 in the absence or presence of E2 for 4 days. The cells were stained with propidium iodide and Annexin V. The rate of apoptosis (A & B) and distribution of the cell cycle (C) were determined using flow cytometry. Click here for file

Additional file 4: Figure S4 E2 enhanced the invasiveness of HCC1428 cells via Gli1. (A) Representative images of wounds at 0 and 48 h in the presence ETOH or E2. (B) Histograms illustrating relative wound widths at 0 and 48 h. The migration distance of each cell was measured after the photographs were converted to Photoshop files. (C) Matrigel invasion assay. HCC1428 cells were seeded into Matrigel-coated invasion chambers and treated with ETOH or E2 for 48 h. Representative images of stained cells are shown. Magnification, 100×. (D) The number of migrated cells was quantified by counting cells from 10 random fields. Data are representative of three independent experiments. Bars represent the means ± SEs of three experiments (**, ##: *P* < 0.01). Click here for file

## References

[B1] Kakarala M Wicha MS Implications of the cancer stem-cell hypothesis for breast cancer prevention and therapy J Clin Oncol 2008 26 2813 2820 10.1200/JCO.2008.16.3931 18539959 PMC2789399

[B2] O'Brien CA Kreso A Jamieson CH Cancer stem cells and self-renewal Clin Cancer Res 2010 16 3113 3120 10.1158/1078-0432.CCR-09-2824 20530701

[B3] Leis O Eguiara A Lopez-Arribillaga E Alberdi MJ Hernandez-Garcia S Elorriaga K Pandiella A Rezola R Martin AG Sox2 expression in breast tumours and activation in breast cancer stem cells Oncogene 2012 31 1354 1365 10.1038/onc.2011.338 21822303

[B4] Jeter CR Liu B Liu X Chen X Liu C Calhoun-Davis T Repass J Zaehres H Shen JJ Tang DG NANOG promotes cancer stem cell characteristics and prostate cancer resistance to androgen deprivation Oncogene 2011 30 3833 3845 10.1038/onc.2011.114 21499299 PMC3140601

[B5] Guo BH Feng Y Zhang R Xu LH Li MZ Kung HF Song LB Zeng MS Bmi-1 promotes invasion and metastasis, and its elevated expression is correlated with an advanced stage of breast cancer Mol Cancer 2011 10 10 10.1186/1476-4598-10-10 21276221 PMC3038148

[B6] Gupta PB Onder TT Jiang G Tao K Kuperwasser C Weinberg RA Lander ES Identification of selective inhibitors of cancer stem cells by high-throughput screening Cell 2009 138 645 659 10.1016/j.cell.2009.06.034 19682730 PMC4892125

[B7] Fillmore CM Kuperwasser C Human breast cancer cell lines contain stem-like cells that self-renew, give rise to phenotypically diverse progeny and survive chemotherapy Breast Cancer Res 2008 10 R25 10.1186/bcr1982 18366788 PMC2397524

[B8] Ginestier C Hur MH Charafe-Jauffret E Monville F Dutcher J Brown M Jacquemier J Viens P Kleer CG Liu S Schott A Hayes D Birnbaum D Wicha MS Dontu G ALDH1 is a marker of normal and malignant human mammary stem cells and a predictor of poor clinical outcome Cell Stem Cell 2007 1 555 567 10.1016/j.stem.2007.08.014 18371393 PMC2423808

[B9] Biddle A Mackenzie IC Cancer stem cells and EMT in carcinoma Cancer Metastasis Rev 2012 31 285 293 10.1007/s10555-012-9345-0 22302111

[B10] Thiery JP Acloque H Huang RY Nieto MA Epithelial-mesenchymal transitions in development and disease Cell 2009 139 871 890 10.1016/j.cell.2009.11.007 19945376

[B11] May CD Sphyris N Evans KW Werden SJ Guo W Mani SA Epithelial-mesenchymal transition and cancer stem cells: a dangerously dynamic duo in breast cancer progression Breast Cancer Res 2011 13 202 10.1186/bcr2789 21392411 PMC3109556

[B12] Mani SA Guo W Liao MJ Eaton EN Ayyanan A Zhou AY Brooks M Reinhard F Zhang CC Shipitsin M Campbell LL Polyak K Brisken C Yang J Weinberg RA The epithelial-mesenchymal transition generates cells with properties of stem cells Cell 2008 133 704 715 10.1016/j.cell.2008.03.027 18485877 PMC2728032

[B13] Barrallo-Gimeno A Nieto MA The Snail genes as inducers of cell movement and survival: implications in development and cancer Development 2005 132 3151 3161 10.1242/dev.01907 15983400

[B14] Zhao J Chen G Cao D Li Y Diao F Cai H Jin Y Lu J Expression of Gli1 correlates with the transition of breast cancer cells to estrogen-independent growth Breast Cancer Res Treat 2010 119 39 51 10.1007/s10549-009-0323-3 19191023

[B15] Cheng G Weihua Z Warner M Gustafsson JA Estrogen receptors ER alpha and ER beta in proliferation in the rodent mammary gland Proc Natl Acad Sci U S A 2004 101 3739 3746 10.1073/pnas.0307864100 14762170 PMC374314

[B16] Clarke RB Howell A Potten CS Anderson E Dissociation between steroid receptor expression and cell proliferation in the human breast Cancer Res 1997 57 4987 4991 9371488

[B17] Harrison H Simoes BM Rogerson L Howell SJ Landberg G Clarke RB Oestrogen increases the activity of oestrogen receptor negative breast cancer stem cells through paracrine EGFR and Notch signalling Breast Cancer Res 2013 15 R21 10.1186/bcr3396 23497505 PMC3672803

[B18] Fillmore CM Gupta PB Rudnick JA Caballero S Keller PJ Lander ES Kuperwasser C Estrogen expands breast cancer stem-like cells through paracrine FGF/Tbx3 signaling Proc Natl Acad Sci U S A 2010 107 21737 21742 10.1073/pnas.1007863107 21098263 PMC3003123

[B19] Mallepell S Krust A Chambon P Brisken C Paracrine signaling through the epithelial estrogen receptor alpha is required for proliferation and morphogenesis in the mammary gland Proc Natl Acad Sci U S A 2006 103 2196 2201 10.1073/pnas.0510974103 16452162 PMC1413744

[B20] Sleeman KE Kendrick H Robertson D Isacke CM Ashworth A Smalley MJ Dissociation of estrogen receptor expression and in vivo stem cell activity in the mammary gland J Cell Biol 2007 176 19 26 10.1083/jcb.200604065 17190790 PMC2063618

[B21] Ruiz IAA Gli proteins encode context-dependent positive and negative functions: implications for development and disease Development 1999 126 3205 3216 10375510 10.1242/dev.126.14.3205

[B22] Kinzler KW Bigner SH Bigner DD Trent JM Law ML O'Brien SJ Wong AJ Vogelstein B Identification of an amplified, highly expressed gene in a human glioma Science 1987 236 70 73 10.1126/science.3563490 3563490

[B23] Yu D Shin HS Lee YS Lee D Kim S Lee YC Genistein attenuates cancer stem cell characteristics in gastric cancer through the downregulation of Gli1 Oncol Rep 2014 31 673 678 24297371 10.3892/or.2013.2893

[B24] Lauth M Toftgard R Non-canonical activation of GLI transcription factors: implications for targeted anti-cancer therapy Cell Cycle 2007 6 2458 2463 10.4161/cc.6.20.4808 17726373

[B25] Ksiazkiewicz M Markiewicz A Zaczek AJ Epithelial-mesenchymal transition: a hallmark in metastasis formation linking circulating tumor cells and cancer stem cells Pathobiology 2012 79 195 208 10.1159/000337106 22488297

[B26] Lin CH Hung PH Chen YJ CD44 is associated with the aggressive phenotype of nasopharyngeal carcinoma through redox regulation Int J Mol Sci 2013 14 13266 13281 10.3390/ijms140713266 23803658 PMC3742186

[B27] Bundred NJ Prognostic and predictive factors in breast cancer Cancer Treat Rev 2001 27 137 142 10.1053/ctrv.2000.0207 11417963

[B28] O'Brien CS Howell SJ Farnie G Clarke RB Resistance to endocrine therapy: are breast cancer stem cells the culprits? J Mammary Gland Biol Neoplasia 2009 14 45 54 10.1007/s10911-009-9115-y 19252972

[B29] O'Brien CS Farnie G Howell SJ Clarke RB Breast cancer stem cells and their role in resistance to endocrine therapy Horm Cancer 2011 2 91 103 10.1007/s12672-011-0066-6 21761332 PMC10358078

[B30] Nicolini A Ferrari P Fini M Borsari V Fallahi P Antonelli A Berti P Carpi A Miccoli P Stem cells: their role in breast cancer development and resistance to treatment Curr Pharm Biotechnol 2011 12 196 205 10.2174/138920111794295657 21044007

[B31] Simoes BM Piva M Iriondo O Comaills V Lopez-Ruiz JA Zabalza I Mieza JA Acinas O Vivanco MD Effects of estrogen on the proportion of stem cells in the breast Breast Cancer Res Treat 2011 129 23 35 10.1007/s10549-010-1169-4 20859678

[B32] Shipitsin M Campbell LL Argani P Weremowicz S Bloushtain-Qimron N Yao J Nikolskaya T Serebryiskaya T Beroukhim R Hu M Halushka MK Sukumar S Parker LM Anderson KS Harris LN Garber JE Richardson AL Schnitt SJ Nikolsky Y Gelman RS Polyak K Molecular definition of breast tumor heterogeneity Cancer Cell 2007 11 259 273 10.1016/j.ccr.2007.01.013 17349583

[B33] Creighton CJ Gibbons DL Kurie JM The role of epithelial-mesenchymal transition programming in invasion and metastasis: a clinical perspective Cancer Manag Res 2013 5 187 195 23986650 10.2147/CMAR.S35171 PMC3754282

[B34] Singh A Settleman J EMT, cancer stem cells and drug resistance: an emerging axis of evil in the war on cancer Oncogene 2010 29 4741 4751 10.1038/onc.2010.215 20531305 PMC3176718

[B35] Goel HL Pursell B Chang C Shaw LM Mao J Simin K Kumar P Vander KC Shultz LD Greiner DL Norum JH Toftgard R Kuperwasser C Mercurio AM GLI1 regulates a novel neuropilin-2/alpha6beta1 integrin based autocrine pathway that contributes to breast cancer initiation EMBO Mol Med 2013 5 488 508 10.1002/emmm.201202078 23436775 PMC3628099

[B36] DiMeo TA Anderson K Phadke P Fan C Perou CM Naber S Kuperwasser C A novel lung metastasis signature links Wnt signaling with cancer cell self-renewal and epithelial-mesenchymal transition in basal-like breast cancer Cancer Res 2009 69 5364 5373 10.1158/0008-5472.CAN-08-4135 19549913 PMC2782448

[B37] Brummelkamp TR Bernards R Agami R A system for stable expression of short interfering RNAs in mammalian cells Science 2002 296 550 553 10.1126/science.1068999 11910072

[B38] Dontu G Abdallah WM Foley JM Jackson KW Clarke MF Kawamura MJ Wicha MS In vitro propagation and transcriptional profiling of human mammary stem/progenitor cells Genes Dev 2003 17 1253 1270 10.1101/gad.1061803 12756227 PMC196056

[B39] Fu J Lv X Lin H Wu L Wang R Zhou Z Zhang B Wang YL Tsang BK Zhu C Wang H Ubiquitin ligase cullin 7 induces epithelial-mesenchymal transition in human choriocarcinoma cells J Biol Chem 2010 285 10870 10879 10.1074/jbc.M109.004200 20139075 PMC2856293

