# Supplementary figures and images for: Estrogen promotes stemness and invasiveness of ER-positive breast cancer cells through Gli1 activation
Source: Mol Cancer. 2014 Jun 3;13:137. doi: 10.1186/1476-4598-13-137 (PMC4057898; doi:10.1186/1476-4598-13-137)

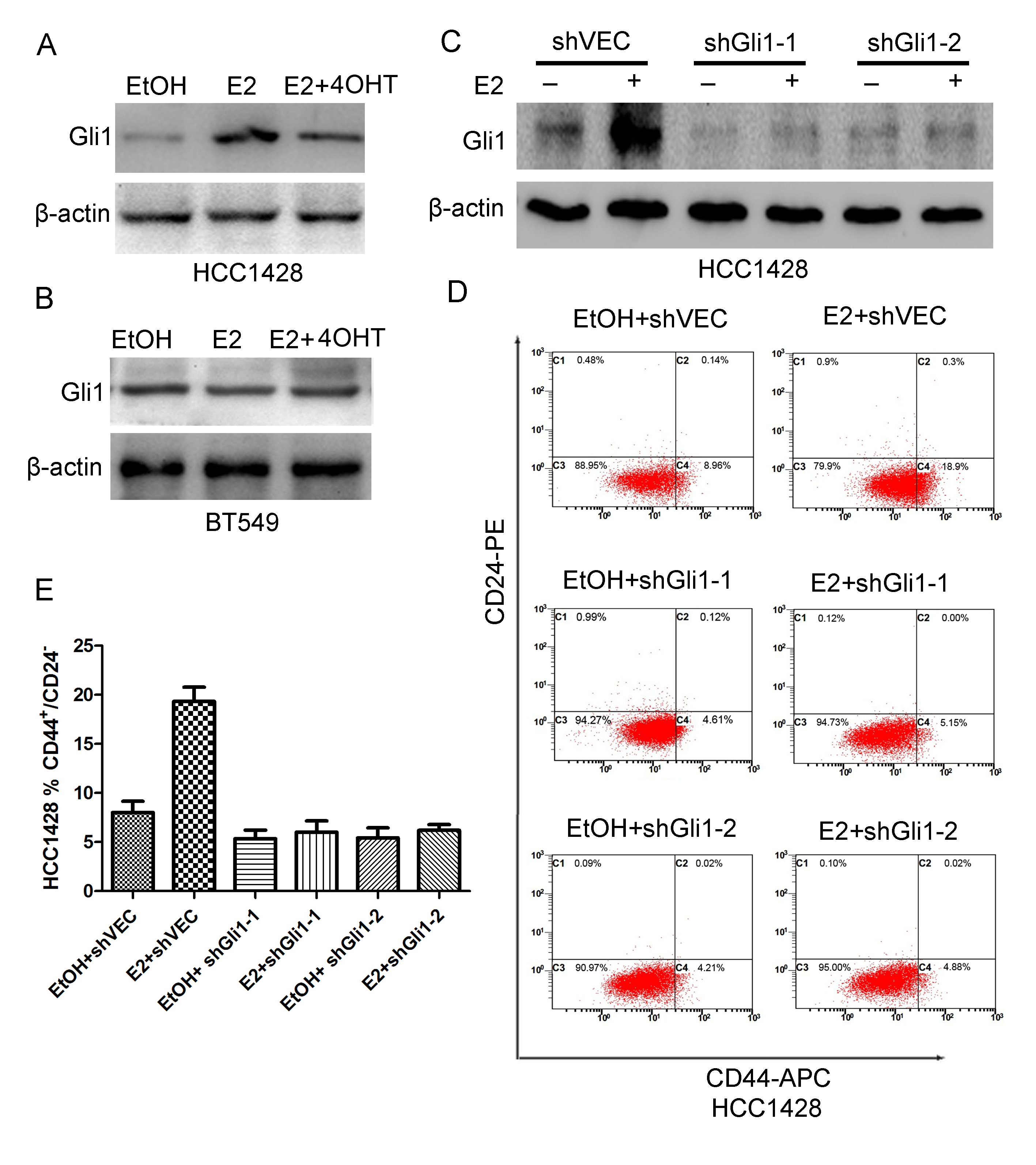

Supplement: Additional file 1: Figure S1 — Estrogen promoted the expression of Gli1 and CSCs in HCC1428 cells. (A & B) Western blotting was used to detect Gli1 in HCC1428 (A) and BT549 (B) cells treated with 10 nM estrogen (E2) with or without 1 μM 4-hydroxy tamoxifen (4OHT) for 4 days. β-Actin was used as a loading control. (C) HCC1428 cells were transfected with control shRNA (shVEC), shGli1-1, or shGli1-2 in the absence or presence of E2. Gli1 protein levels were analyzed by western blotting, and β-actin levels were measured as a loading control. (D) HCC1428 cells were treated with 10 nM E2 or ETOH (control) and transfected with shGli1-1, shGli1-2, or shVEC. After 4 days, cells were stained with anti-CD44-APC and anti-CD24-PE antibodies, and CD44+/CD24-/low subpopulations were examined by flow cytometry. (E) Histograms illustrating the percentage of CD44+/CD24-/low subpopulations. All data correspond to the mean ± SD of three independent experiments. **, ## indicate significantly different from the control, p < 0.001. [file 1476-4598-13-137-S1.jpeg]

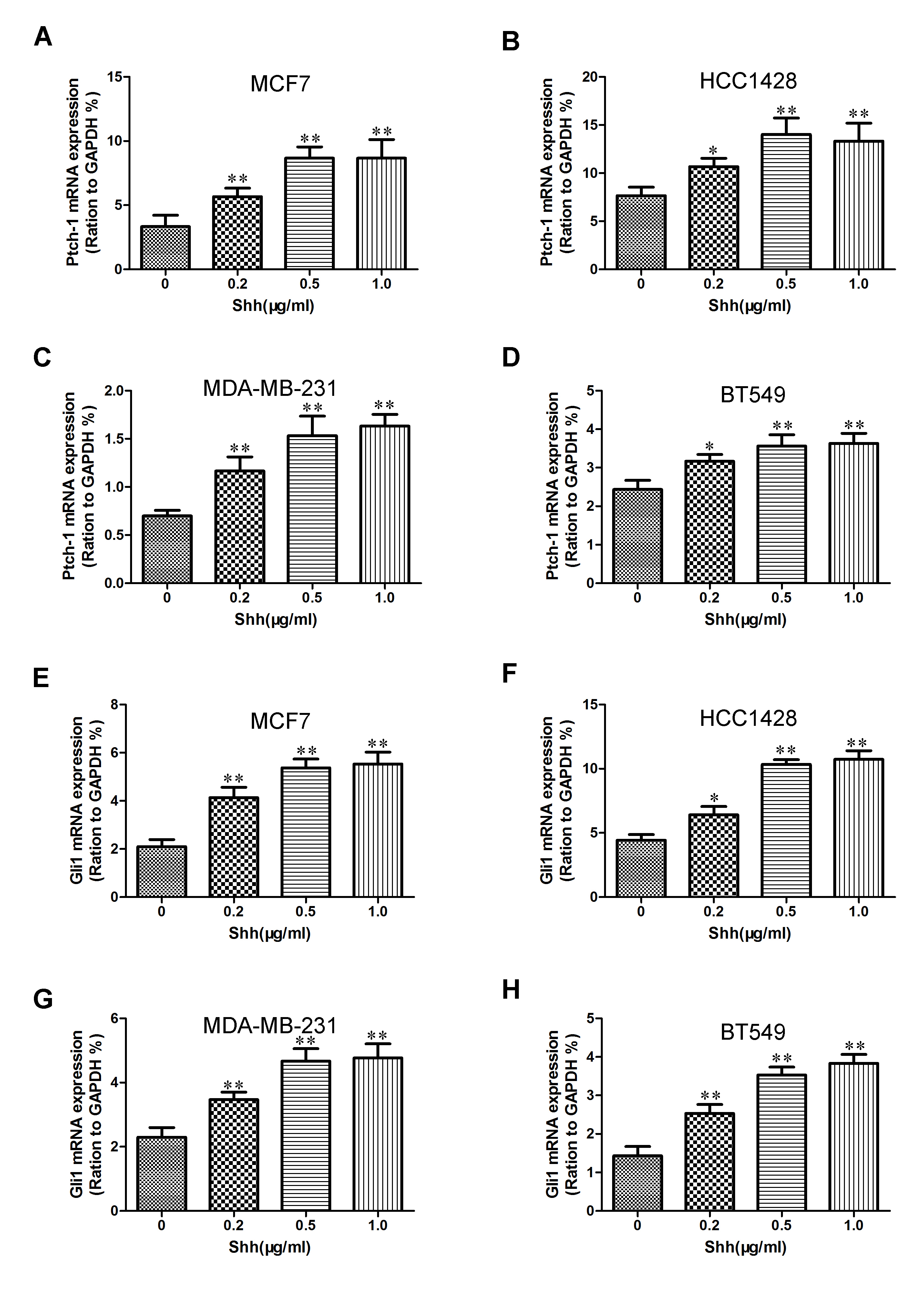

Supplement: Additional file 2: Figure S2 — Effects of Shh on Ptch1 and Gli1 mRNA expression in breast cancer cells. Shh was added at the specified levels to serum-starved breast cancer cells for 24 h, after which total RNA was extracted and subjected to qRT-PCR analysis for Ptch1 and Gli1 mRNAs expression levels. qRT-PCR was used to detect Ptch1 in MCF7 (A), HCC1428 (B), MDA-MB-231 (C), and BT549 (D). qRT-PCR was used to detect Gli1 in MCF7 (E), HCC1428 (F), MDA-MB-231 (G), and BT549 (H). All data correspond to the mean ± SD of three independent experiments. *indicate significantly different from the control, p < 0.05. **indicate significantly different from the control, p < 0.01. [file 1476-4598-13-137-S2.jpeg]

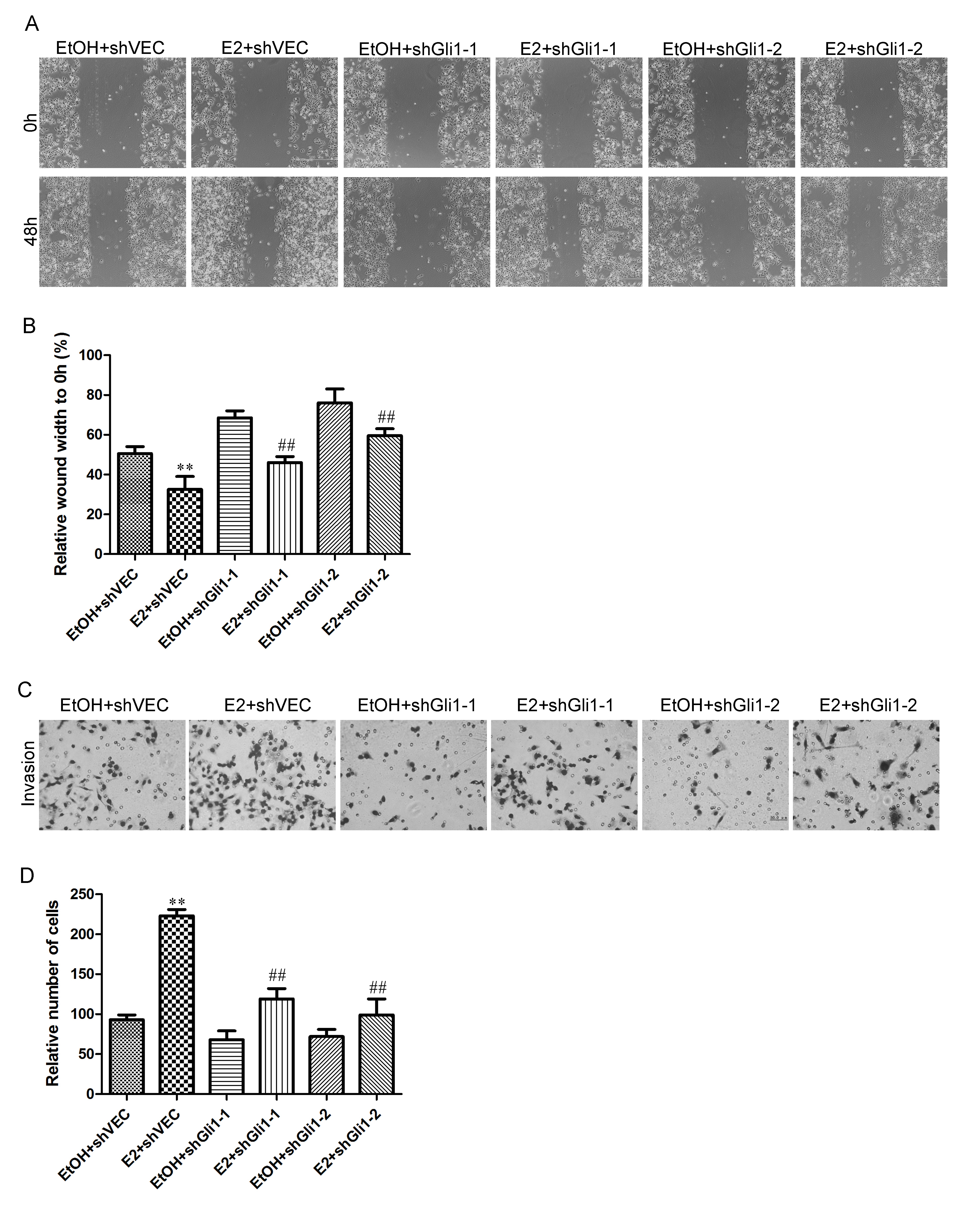

Supplement: Additional file 3: Figure S3 — Effects of E2 on the cell cycle and apoptosis of shVEC- and shGli1-transfected MCF-7 cells. MCF-7 cells were transfected with control shRNA (shVEC), shGli1-1, or shGli1-2 in the absence or presence of E2 for 4 days. The cells were stained with propidium iodide and Annexin V. The rate of apoptosis (A & B) and distribution of the cell cycle (C) were determined using flow cytometry. [file 1476-4598-13-137-S4.jpeg]

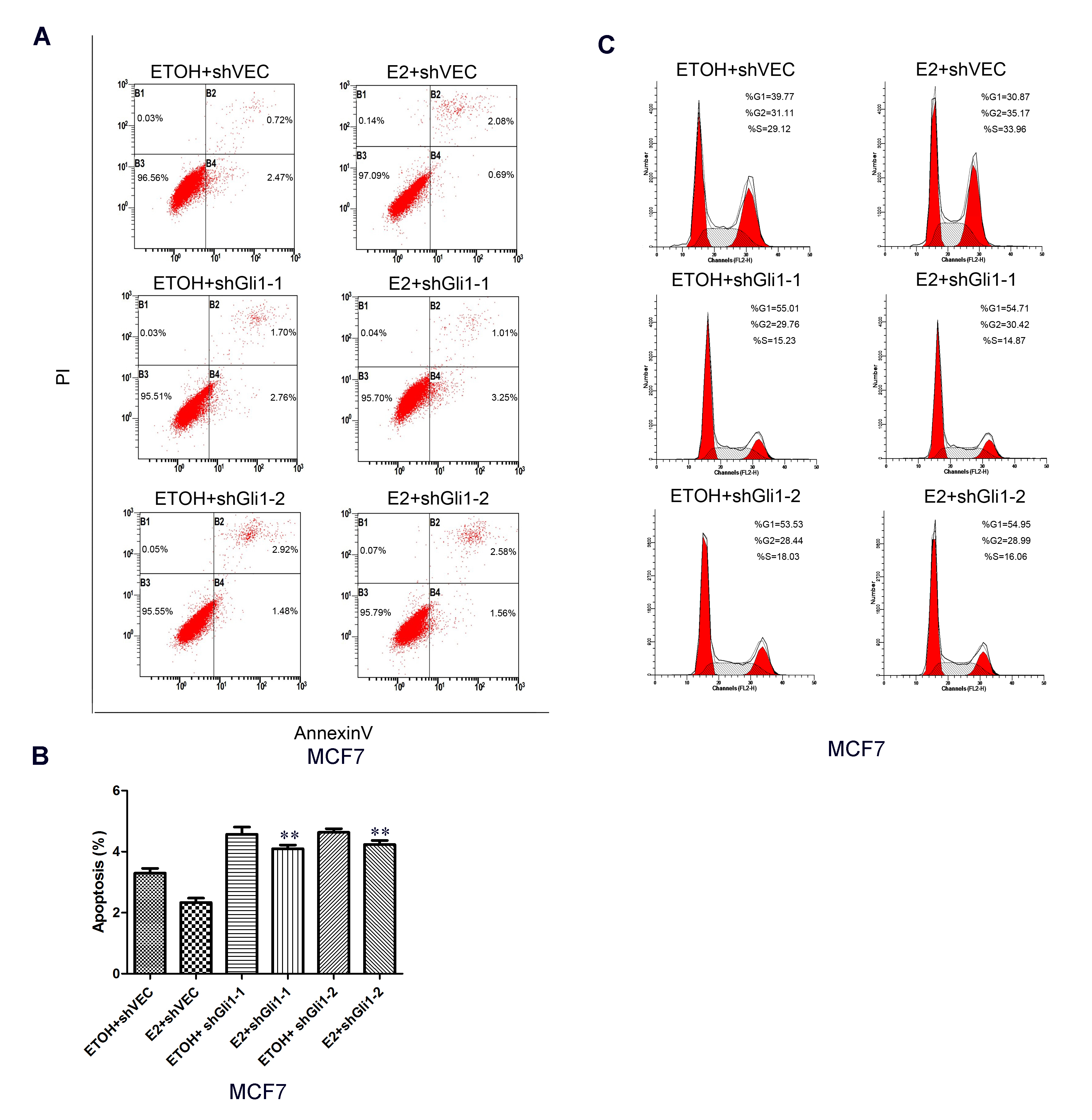

Supplement: Additional file 4: Figure S4 — E2 enhanced the invasiveness of HCC1428 cells via Gli1. (A) Representative images of wounds at 0 and 48 h in the presence ETOH or E2. (B) Histograms illustrating relative wound widths at 0 and 48 h. The migration distance of each cell was measured after the photographs were converted to Photoshop files. (C) Matrigel invasion assay. HCC1428 cells were seeded into Matrigel-coated invasion chambers and treated with ETOH or E2 for 48 h. Representative images of stained cells are shown. Magnification, 100×. (D) The number of migrated cells was quantified by counting cells from 10 random fields. Data are representative of three independent experiments. Bars represent the means ± SEs of three experiments (**, ##: P < 0.01). [file 1476-4598-13-137-S3.jpeg]
